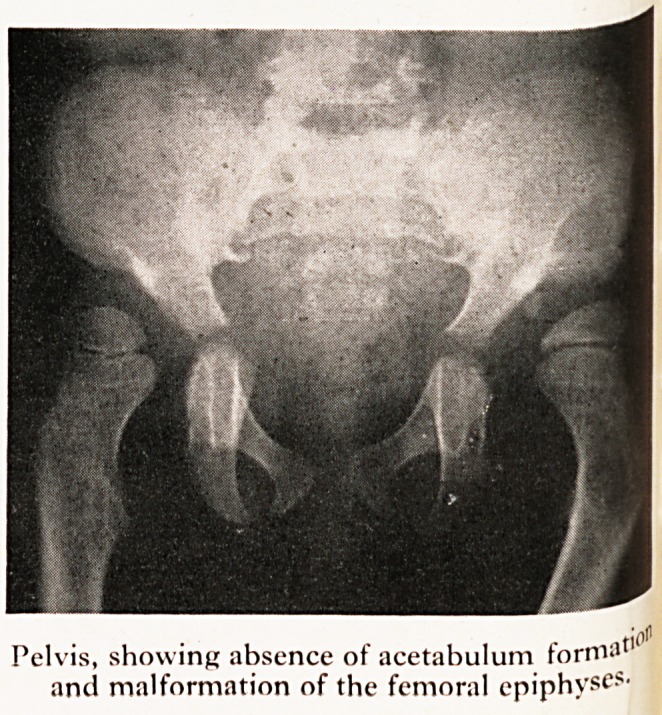# Gargoylism

**Published:** 1952-07

**Authors:** C. R. Croft

**Affiliations:** Physician, Plymouth, South Devon and East Cornwall Hospital


### GARGOYLISM

BY

C. R. CROFT, D.M., M.R.C.P.

*Physician, Plymouth, South Devon and East Cornwall Hospital*

Lindsay (1950) reported that 156 cases of this disease had been described. It is therefore not common, but among mentally defective children there are probably many more who belong to this group. Although no patient will benefit from this diagnosis at the present time, its more frequent recognition may bring a better understanding of the disease. It is with this hope that a further case is described.

*John B.* was born on the 2nd March, 1948, being delivered by the breech. His parents thought well of him, but within two months he was found to have double inguinal herniae. He also had trouble with feeding, which yielded to the bottle.

*At 7 months*, it was noticed that his back was weak. X-ray pictures showed kyphosis, scoliosis and congenital dislocation of the left hip but no other evidence of abnormal development or bone disease. The inguinal herniae were repaired shortly after his first birthday, and he was circumcised at the same time. At this time squareness of the head and prominence of the frontal bones were noted, together with slight enlargement of the liver.

*At 2½ years* he was clearly mentally defective, and he was regarded as a mongol. He was unable to say more than a few words but he responded to simple requests; he could feed himself and was clean in his habits. He walked steadily in a waddling manner. He was a large but stocky child and steadily increasing in weight. In addition to kypho-scoliosis he now had bilateral dislocation of the hips and an umbilical hernia, for which he was treated surgically.

*At 3½ years* his abdomen began to increase in size and his stools became rather loose. His appearance was now remarkable. He had a large square uncouth head, with heavy features. His eyes were rather wide apart, and the bridge of his nose depressed, but his eyes were lively and his expression amiable. His head was sunk squarely between wide high shoulders (Plate II). His trunk was squat and solid, and supported by a firm stance with his feet wide apart. Other peculiarities were now observed. His corneae were hazy, his skin thick and rather dry, his hands and feet were large, and the bones of his forearms and legs thickened. Dorsal kyphosis was present, and his thoracic cage seemed too large for him. His liver reached three inches below his ribs and was smooth. Blood Wassermann reaction was negative.

On X-ray examination abnormalities were seen in almost every bone in his body (Plate III). In his rather large skull the most characteristic change was an elongated, boat-shaped, pituitary fossa, without any sign of erosion of the clinoid processes. The mandible was massive. There was dorsal kypho-scoliosis, while the second lumbar vertebra and, to less extent, the first and third showed the anterior " beaking " of the vertebral body, which is characteristic of the disease. The upper and lower surfaces of the vertebral bodies were convex, so that they presented a somewhat circular outline. The ribs were very wide. The changes in both hip joints were similar to those of congenital dislocation, with defective development of the heads of the femora. The bones of the limbs, particularly of the forearms, fingers and legs were widened with some loss of the normal bony texture.

### DISCUSSION

Although this condition was recognized in 1900, the first adequate description was by Hunter (1917), who was then a major in the Canadian Army

PLATE II

Gargoylism, showing the heavy square head implanted between the wide shoulders. Also the stance, the big thorax, and the enlarged belly with huge liver. The mark to the left of the liver indicates the left costal margin.

X-ray of spine, showing the convex upper and lower surfaces of the vertebrae and the anterior " beaking " of the upper lumbar vertebral bodies, especially the second.

Right arm and metacarpus, showing thickening of long bones and loss of bony structure.

Skull, showing elongated pituitary fossa.

Pelvis, showing absence of acetabulum formation and malformation of the femoral epiphyses.

Medical Corps. Hurler published two cases in 1919, and it became known as Hurler's Syndrome, but in 1935 she suggested the name dysostosis multiplex. Ellis (1936) christened it gargoylism, by which term it is most commonly known; but, in 1937, Washington suggested lipochondrodystrophy.

It is slightly more common in boys than in girls, and there is a familial tendency which is sufficiently strong to establish a genetic factor in the causation (Jervis, 1950). Although it may be present at birth it is seldom recognized under one year of age. Most patients die before their tenth year; a few survive to fifteen, and occasionally to twenty years.

This case illustrates all the gross changes which have been described in the disease except for splenic enlargement. The osseous changes are more fully described by Fairbank (1949). It remains therefore to discuss the curious cellular changes underlying the gross pathology. From the scanty reports on histology it seems that every tissue in the body is infiltrated by an intracellular and extracellular deposit. This affects mainly the cardio-vascular, skeletal, reticulo-endothelial, nervous, and endocrine systems. The heart and blood vessels are particularly affected, the ventricles being enlarged and the vessels thickened; these changes are the commonest cause of death (Lindsay, 1950). Opinions differ as to the nature of the deposit. Lindsay (1948, 1950) considers that the substance is glycogen, probably in combination with protein, but Green (1948) and Jervis (1950) regard it as a lipoid substance. The evidence in favour of glycogen is based mainly on biopsy, and that in favour of lipoid on post-mortem material, in which glycolysis may well have occurred. Despite these doubts it is generally agreed that gargoylism should be placed in the group of storage diseases. In these various diseases, the macromolecular substance in the tissues, although varied in constitution and location, may produce similar physiological changes.

Diagnosis is not difficult if the condition is remembered. The only likely alternative is congenital syphilis, and this should be excluded. The onset of acromegaly may be suspected. In the type of dwarfism known as the Morquio-Brailsford syndrome there is a somewhat similar kyphosis, but radiological appearances of the spine are distinctive and true " beaking " of the vertebrae is not seen; moreover, mental deficiency and corneal opacity are absent.

Cretinism, or later mongolism, may be suspected, but superficial examination is sufficient to exclude these. Many *formes frustes* are described, usually in siblings of well-established cases, and in these the diagnosis may present great difficulty and only be confirmed by microscopy (Jervis, 1950).

Corneal opacity and hepato-splenomegaly are not essential for diagnosis, but mental defect is almost invariably present, together with the peculiar and characteristic facies, dwarfism, and skeletal deformity, particularly of the spine. The facies is of paramount importance in arousing suspicion of the disease, and in all such cases spinal X-ray pictures should be examined. There is remarkable similarity in the published photographs of these patients, and their appearance fully justifies the retention of the name " gargoylism ".

This possible diagnosis should be borne in mind in examining all mentally-defective children with bony deformities, or even with herniae. When the effect of the disease upon the cardio-vascular system is appreciated there may be less readiness to regard them as cases for routine surgery. In all doubtful cases in which surgery is undertaken, biopsy should be carried out and for this purpose circumcision may suffice.

I am very grateful to Dr. T. L. Chester-Williams for the photographs, and to Dr. A. Craig Mooney for the use of the X-ray films.

### REFERENCES

Ellis, R. W. B., Sheldon, W., and Capon, N. B. (1936). *Quart. J. Med.* **29**, **5**, 119.
Fairbank, H. A. T. (1949). *J. Bone and Jt. Surg.* **31** *b*, 291.
Green, M. A. (1948). *J. Neuropath and Exp. Neurology*, **7**, 399.
Hunter, C. (1917). *Proc. Roy. Soc. Med.* **10**, 104, Sect. Stud. Dis. Child.
Hurler, G. (1919). *Zeits. J. Kinderheilk.* **24**. 220.
Washington, J. A. (1937). Lipochondrodystrophy, in Brennermann, *J. Practice of Pediatrics*, Hagerstoun, Md., W. F. Prior, Inc. 1937, vol. iv, chap. 31.
Hurler, J. (1935). Degenerative Dysostosis, in von Pflaundler, M. and Schlossman, A.: *Diseases of Children*, translated by M. G. Peterman. Philadelphia, J. B. L. Lippincott Co., 1935, vol ii, p. 29.
Jervis, G. A. (1950). *Arch. Neurology and Psychiatry*, **63**, 681.
Lindsay, S. (1948). *Amer. J. Dis. Child.*, **76**, 239.
Lindsay, S. (1950). *British Heart Journal* **12**, 17.

## Figures and Tables

**Figure f1:**
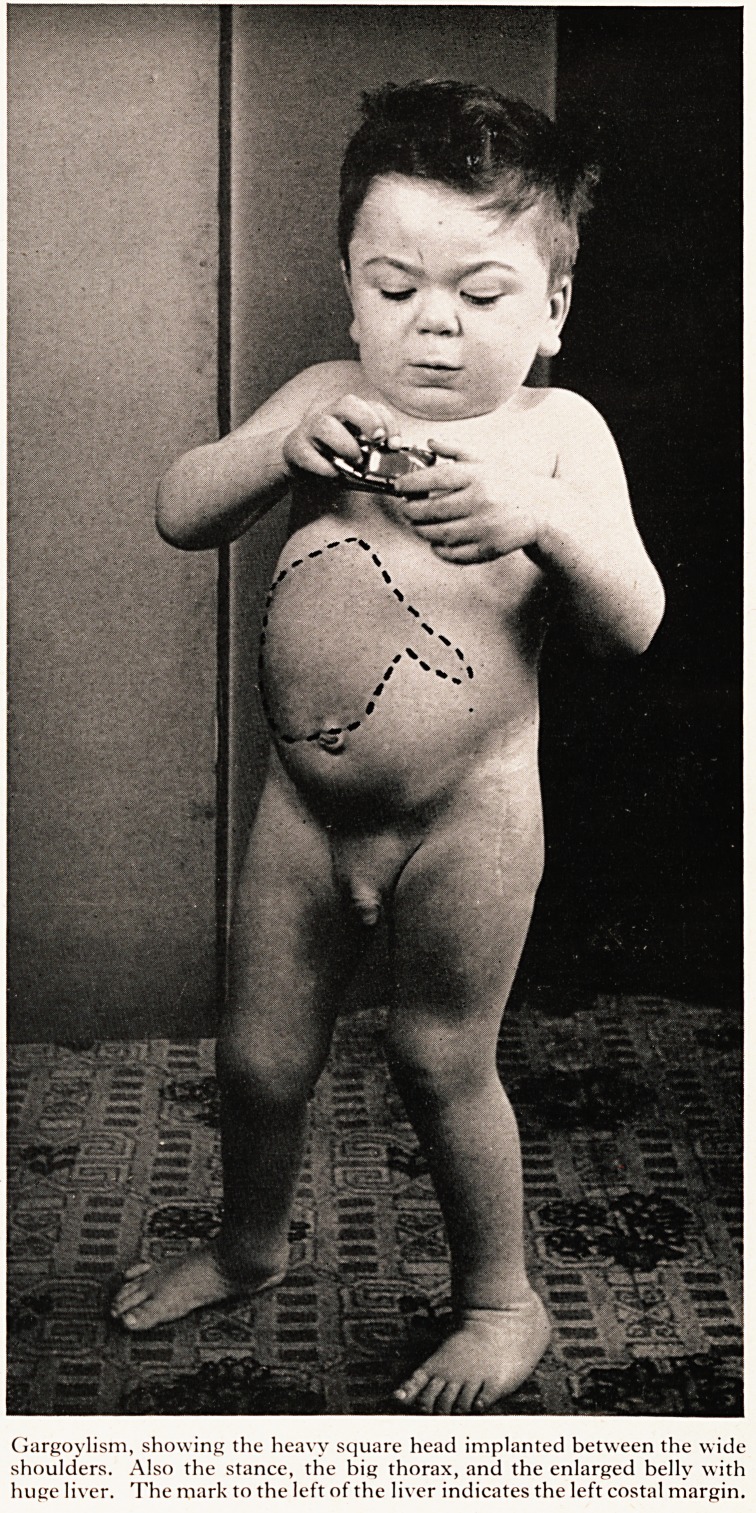


**Figure f2:**
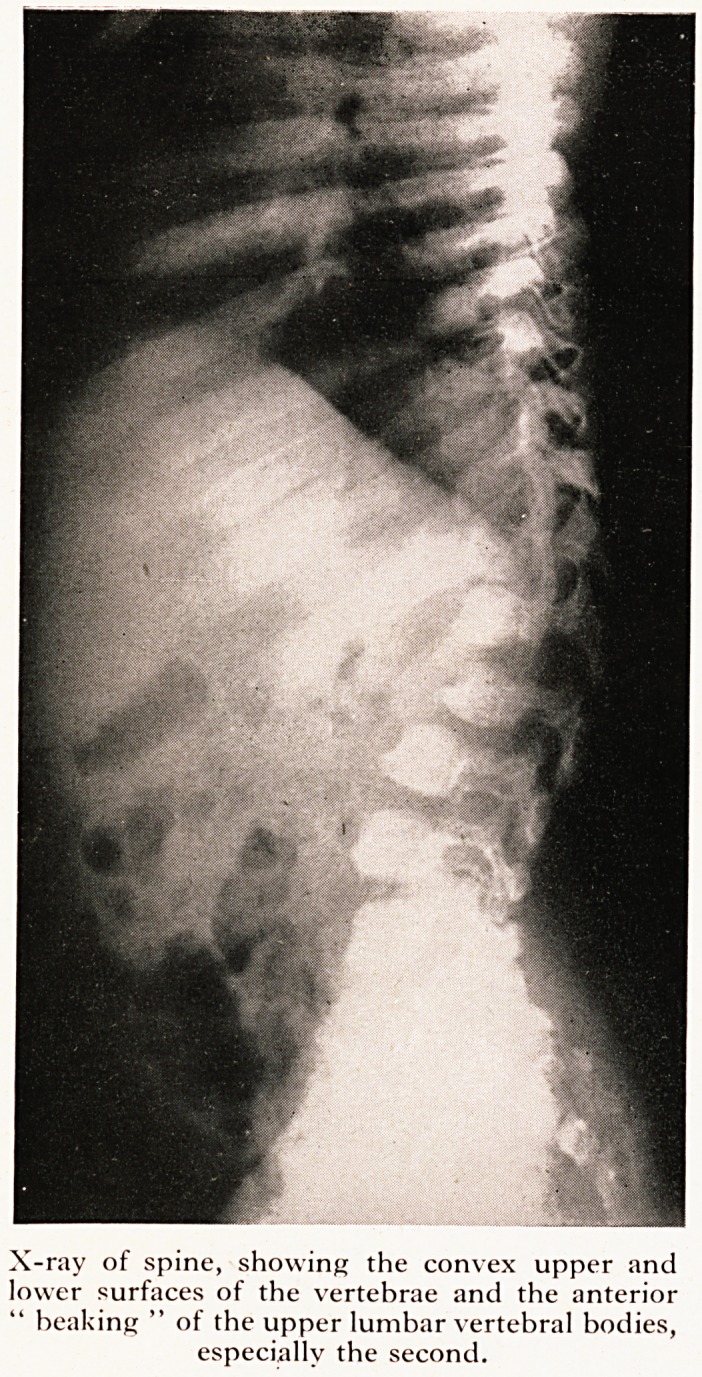


**Figure f3:**
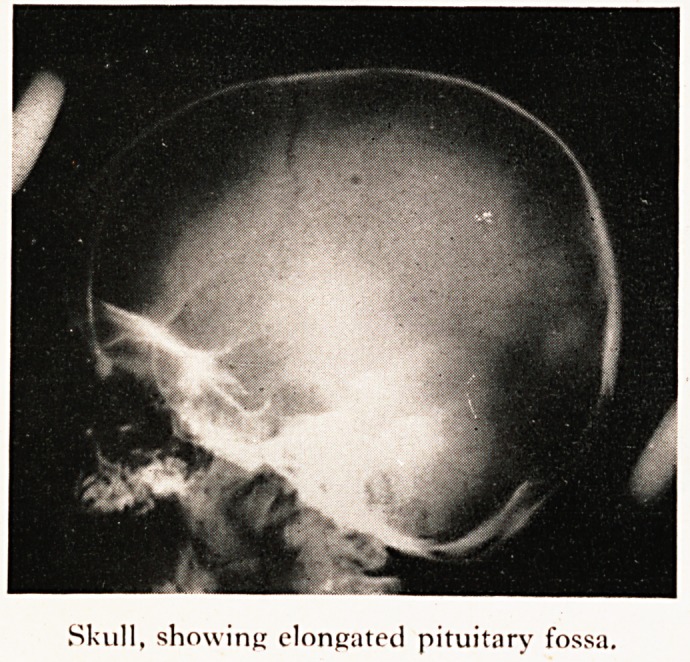


**Figure f4:**
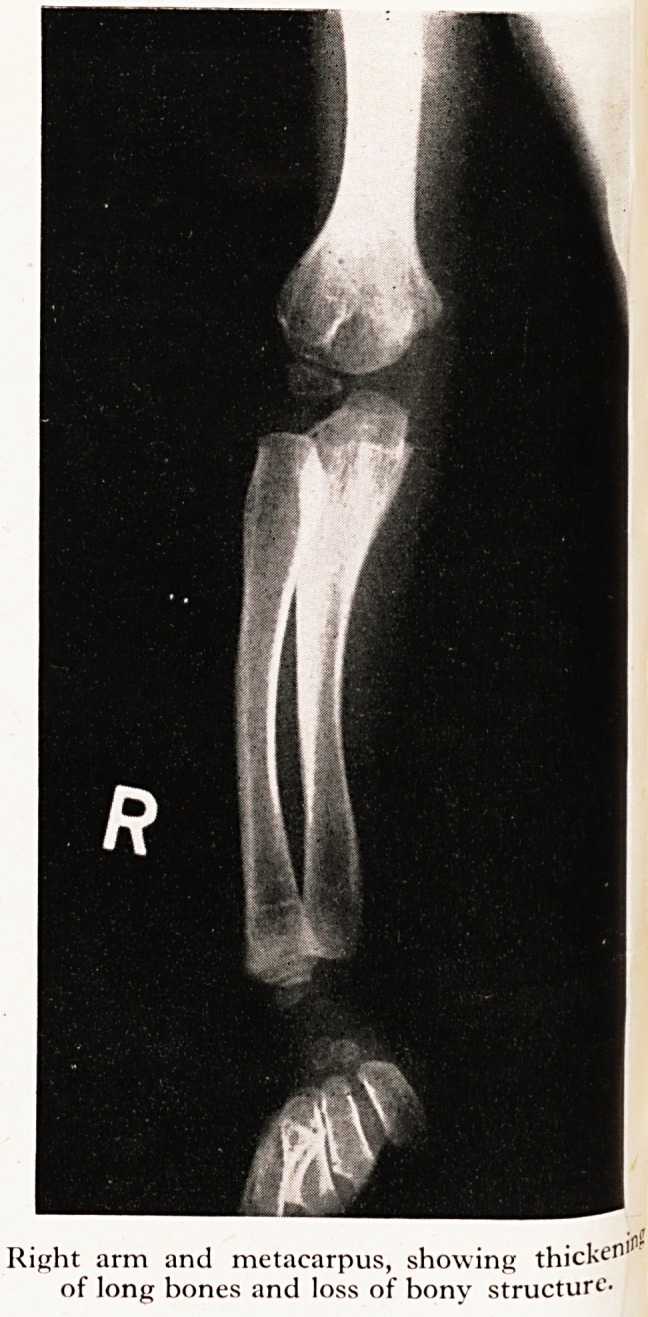


**Figure f5:**